# Dr. Elliott H. Woolsey

**Published:** 1907-02

**Authors:** 


Dr. Elliott H. Woolsey, of Oakland, Cal., a graduate of the University of Buffalo, 1868, died at his home January 21, 1907, of pneumonia, aged 65 years. He was a member of the Society of Physicians and Surgeons of Alameda County, of the Medical Society of the State of California, and of the American Medical Association. He was also for several years chief surgeon of the Southern Pacific Railway.

